# Increased Specific Labeling of INS-1 Pancreatic Beta-Cell by Using RIP-Driven Cre Mutants with Reduced Activity

**DOI:** 10.1371/journal.pone.0129092

**Published:** 2015-06-05

**Authors:** Gen-cheng Gong, Wen-zhu Fan, Di-zheng Li, Xiong Tian, Shao-jun Chen, Yu-cai Fu, Wen-can Xu, Chi-ju Wei

**Affiliations:** 1 Multidisciplinary Research Center, Shantou University, Shantou, Guangdong, 515063, China; 2 Laboratory of Cell Senescence, Shantou University Medical College, Shantou, Guangdong, 515041, China; 3 Department of Endocrinology, the First Affiliated Hospital of Shantou University Medical College, Shantou, Guangdong, 515041, China; NIDCR/NIH, UNITED STATES

## Abstract

Ectopically expressed Cre recombinase in extrapancreatic tissues in RIP-Cre mice has been well documented. The objective of this study was to find a simple solution that allows for improved beta-cell specific targeting. To this end, the RIP-Cre and reporter CMV-loxP-DsRed-loxP-EGFP expression cassettes were configurated into a one-plasmid and two-plasmid systems, which labeled approximately 80% insulin-positive INS-1 cells after 48 h transfection. However, off-target labeling was robustly found in more than 15% insulin-negative Ad293 cells. When an IRES element was inserted in front of Cre to reduce the translation efficiency, the ratio of recombination between INS-1 and Ad293 cells increased 3-4-fold. Further, a series of Cre mutants were generated by site-directed mutagenesis. When one of the mutants, Cre(H289P) in both configurations, was used in the experiment, the percentage of recombination dropped to background levels in a number of insulin-negative cell lines, but decreased only slightly in INS-1 cells. Consistently, DNA substrate digestion assay showed that the enzymatic activity of Cre(H289P) was reduced by 30-fold as compared to that of wild-type. In this study, we reported the generation of constructs containing RIP and Cre mutants, which enabled enhanced beta-cell specific labeling *in vitro*. These tools could be invaluable for beta-cell targeting and to the study of islet development.

## Introduction

Over the past decade, the adoption of the Cre/loxP system for genetic manipulation has impinged profoundly on the study of pancreatic islet development and beta-cell function [1–3]. Various promoters including that of the pancreas duodenal homeobox 1 (Pdx1) [4,5], neurogenin 3 (Ngn3) [6] and insulin (Ins1 and Ins2) [7,8] genes have been used to confine the expression of Cre recombinase within the pancreas populations, and among these, the rat Ins2 promoter (RIP) was the most commonly chosen regulatory sequence for generating the transgenic model [1]. Results from these mice demonstrate that pdx1-expressing precursors give rise to all pancreatic lineages [4], and that different types of endocrine cells in the islets of Langerhans are derived from ngn3-expressing progenitors [6]. In order to trace the fate of post mitotic islet beta-cells *in vitro*, Russ *et al*. produced two lentiviral vectors, one contains the RIP-Cre cassette, the other encompasses the reporter CMV-loxP-DsRed-loxP-EGFP construct [9]. Their findings provide direct evidences that terminally differentiated human islet beta-cells are capable of dedifferentiation and proliferation.

However, numerous studies have demonstrated that many widely used Cre mice are not as tissue-specific as previously intended [10–12]. For example, the supposedly pancreas-specific transgenic Cre drivers show ectopic expression, although mainly in the brain, raising concern of whether the resultant phenotype, when cross to the reporter line, can be completely attributed to alterations in the pancreas [13,14]. This is consistent with the reports that insulin expression can be detected, albeit at low levels, in the brain and other extra-pancreatic organs under physiological and pathological conditions [15,16]. Further, toxicity and glucose intolerance have been found in RIP-Cre mice in the absence of genes targeted by loxP sites [17,18]. In line with this, a genome-wide survey has identified a myriad of cryptic loxP sites in mammalian cells that can promote unintended DNA recombination and damage [19].

In this study, we proposed a simple solution to improve beta-cell specific targeting by using Cre mutants with reduced enzymatic activity. The RIP-Cre cassette and the floxed reporter were configurated into a one-plasmid or two-plasmid system. Transient transfection into insulin-positive and-negative cells allowed easy and sensitive detection of non-specific targeting.

## Materials and Methods

### Plasmid construction

**Fig 1:**
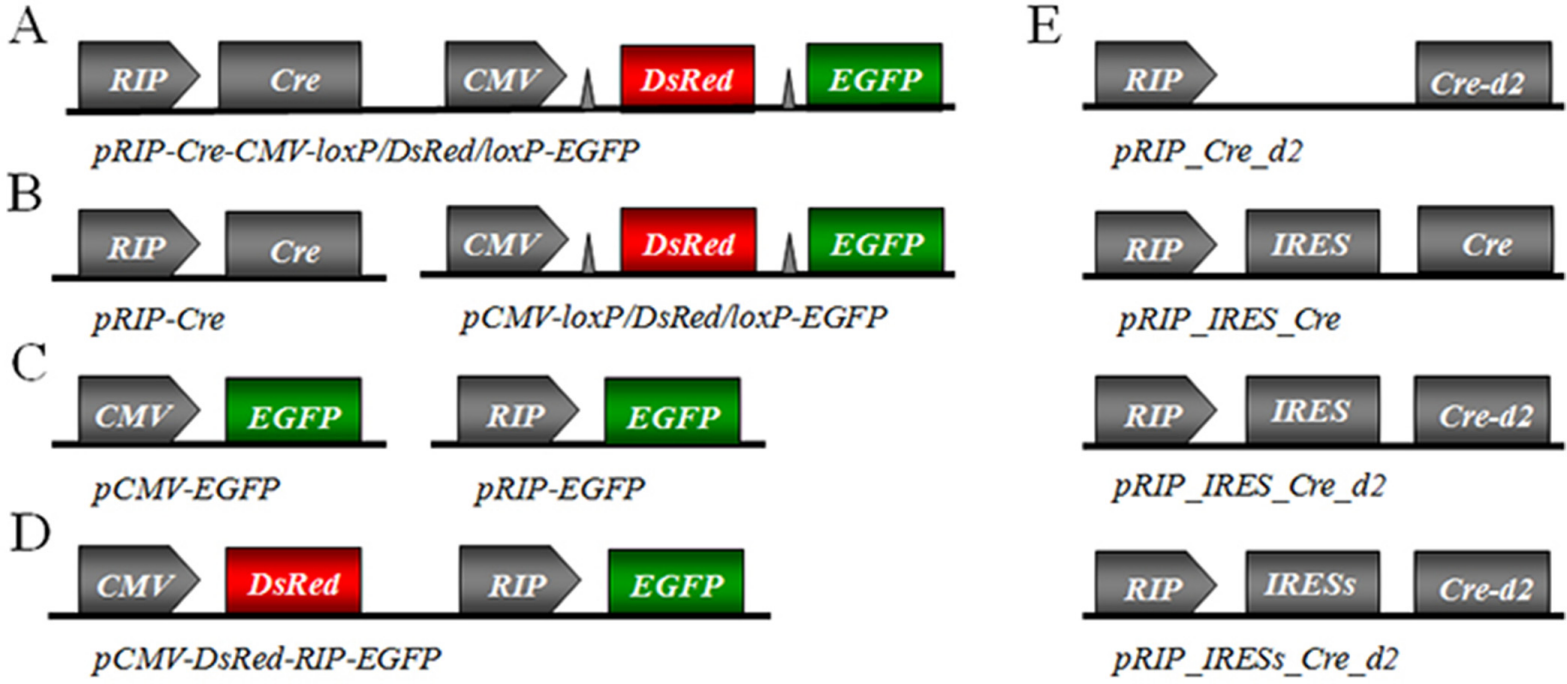
Schematic diagram of plasmids constructed in this study. (A) One plasmid system. (B) Two plasmid system. In these designs, when Cre recombinase is expressed, DsRed will be deleted, while EGFP will be activated. (C) Plasmids for testing RIP specificity. (D) Plasmid for testing promoter interference. If promoter interference exists, insulin-negative cells should turn green; otherwise they are red-only. (E) Plasmids with reduced Cre expression levels. The d2 sequence promotes accelerated degradation of Cre fusion protein; while the insertion of IRES reduces the translation efficiency of Cre. The triangles in (A) and (B) represent loxP sites. Each coding sequence has a polyadenylation site in behind.

All plasmids were generated from pEGFP-N1, pDsRed-Express-1, pd2EYFP-N1, pIRES2 of Clontech (Mountain View, CA), pRIP-CreER [20] of Addgene (Cambridge, MA), pUC18 of Life Technologies (Rockvile, MD), and p210 [21] containing an intron-bearing Cre recombinase (generous gift from Dr. J.E. Green) using standard cloning techniques as described below, and verified by sequencing by Jinsirui (Nanjing, China). A schematic diagram of constructed plasmids was shown in Fig 1.

For all PCR reactions except site-directed mutagenesis, the following conditions were used: 200–500 pg of DNA template, 1 mM MgSO4, 0.3 mM dNTPs, 0.3 μM primers, and 1.25 U of Pfu-polymerase (Life Technologies, Rockvile, MD) in a total reaction volume of 50 μl. Thermocycle parameters were: 98°C for 2 min, followed by 98°C for 10 s, 60–65°C for 5 s and 72°C for 1 kb/min for a total of 35 cycles. Sequences of primer sets were listed in Table 1.

**Table 1:** PCR primer sets used for plasmid construction.

	F-primer	R-primer
1	TATCTTAAGTGCGTTATCCCCTG	CGCTTACAATTTACGCCTTAAG
2	CATCTGCCACCAGCCAGCTATCA	TTAAAAGCTTATCGCCATCTTCCAGCA
3	CGATAAGCTTAGCCATGGCTT	ACGCCTTAAGAATTCGATGAG
4	TCTATATCTGCAGGCGCGCGGT	ATCCGCCGCATAACCAGTGAAAC
5	CGTCGTCCTCCTCGCTTG	TAGGTAGAGCGCGGCGA

### Generation of the two-plasmid system

#### Construction of pRIP-Cre

The intron-bearing Cre recombinase was cut with KpnI/NotI from p210 [21], and used to replaced the CreER fragment (KpnI/NotI) of pRIP-CreER [20].

#### Construction of pCMV-loxP/DsRed/loxP-EGFP

A DNA fragment containing two loxP sites in tandem repeat and an EcoRV site in between was synthesized by Yingjun (Shanghai, China). Also, the fragment was designed to contain two sticky 3*′*-ends compatible with HindIII and SacII sites, respectively. After phosphrylation at 5*′*-ends, the fragment was ligated into pUC18 at HindIII/SacII sites. The DsRed fragment containing a polyadenylation site was cut with AgeI/AflII from pDsRed-Express-1, blunt-ended and ligated to pUC-loxP/loxP at EcoRV. The loxP-DsRed-loxP fragment was then cut with HindIII/BamHI, and ligated to pEGFP-N1 at HindIII/BamHI, finishing the reporter construct pCMV-loxP-DsRed-loxP-EGFP.

### Generation of the one-plasmid system

#### Construction of pRIP-Cre-CMV-loxP/DsRed/loxP-EGFP

The CMV-loxP-DsRed- loxP-EGFP fragment was PCR amplified from the reporter plasmid with primers F1/R1 containing AflII at both 5*′*-ends, and then ligated into pRIP-Cre at AflII site.

### Generation of plasmids for promoter specificity and interference

#### Construction of pRIP-EGFP

A Kpn/NotI fragment containing the EGFP sequence was cut from pEGFP-N1, and then used to replace the CreER (Kpn/NotI) fragment from pRIP-CreER.

#### Construction of pCMV-DsRed-RIP-EGFP

The DsRed fragment containing a polyadenylation site was cut with BamHI/AflII from pDsRed-Express-1, blunt-ended and ligated to pEGFP-N1 at BamHI/AflII. The EGFP fragment (BamHI/AflII) from pEGFP-N1 was ligated to pRIP-CreER at BamHI/AflII. A BglII/AflII fragment containing the RIP-EGFP-polyA sequence was cut from pRIP-EGFP, blunt-ended, and inserted after the DsRed sequence of pCMV-DsRed at AflII site.

### Generation of plasmids with reduced Cre protein levels

#### Construction of pRIP-Cre-d2

Three pieces ligation was performed to construct pRIP-Cre-d2. The C-terminus of Cre was amplified from pRIP-Cre with primers F2/R2 containing ClaI and HindIII at 5*′*-ends, respectively. The PEST fragment of the murine ornithine decarboxylase (d2), which stimulates protein degradation, was amplified from pd2EYFP-N1 with primers F3/R3 containing HindIII and AflII at 5*′*-ends, respectively. Finally, pRIP-Cre was cut with ClaI and AflII, and ligated with the PCR amplified Cre C-terminal and d2 fragments.

#### Construction of pRIP-IRES-Cre

Three pieces ligation was performed to construct pRIP-IRES-Cre. The IRES fragment was cut with MluI and SalI from pIRES2. The Cre fragment was cut with XhoI and AflII from pRIP-Cre. The sticky ends of SalI and XhoI are compatible. Finally, pRIP-Cre was cut with MulI and AflII, and ligated with the IRES and Cre fragments.

#### Construction of pRIP-IRES-Cre-d2

Three pieces ligation was performed to construct pRIP-IRES-Cre. The IRES fragment was cut with MluI and SalI from pIRES2. The Cre-d2 fragment was cut with XhoI and AflII from pRIP-Cre-d2. Finally, pRIP-Cre-d2 was cut with MulI and AflII, and ligated with the IRES and Cre fragments.

#### Construction of pRIP-IRESs-Cre-d2

pRIP-IRES-Cre-d2 was cut with XmaI between IRES and Cre, blunt-ended and religated. A frame shift was created between IRES and Cre, and therefore further degreasing the translation efficiency.

### Site-directed mutagenesis

Site-directed mutagenesis was performed by PCR with one of the primers carrying point mutation at the 5*′*-end. Sequences of primer sets were listed in Table 2. Plasmid DNA was diluted with ddH_2_O to 400 pg/μl, and the following conditions were used for PCR according to the instruction of the company: 1 μl of DNA template, 1 μl primers mix (10 mM), 25 μl PrimeSTAR mix (Takara, Dalian, China) in a total reaction volume of 50 μl. Thermocycle parameters were: 98°C for 2 min, followed by 98°C for 10 s, 50–65°C for 5 s and 72°C for 5 min for a total of 30 cycles. Purified PCR product was phosphrylated at 5*′*-end and ligated for transformation into *E*. *coli* TOP10.

**Table 2:** PCR primer sets used for site-directed mutagenesis.

Mutat.	F-primer	R-primer
R173A	GTTAGCTATAGCCGAAATTGCCAG	AGGGTGTTATAAGCAATCCCCAG
R173H	ACATATAGCCGAAATTGCCAG	AACAGGGTGTTATAAGCAATCC
R173K	GTTAAAGATAGCCGAAATTGCCAG	AGGGTGTTATAAGCAATCCCCAG
R173L	GTTACTTATAGCCGAAATTGCCAG	AGGGTGTTATAAGCAATCCCCAG
E176F	ATAGCCTTCATTGCCAGGATCAG	ACGTAACAGGGTGTTATAAGCAATCC
E176V	ATAGCCGTAATTGCCAGGATCAG	ACGTAACAGGGTGTTATAAGCAATCC
K201R	GAACGAGGACGCTGGTTAGC	TGCCAATATGGATTAACATTCTCC
H289F	ATTCAGTGCCCGTGTCGGAG	CCAGACCAGGCCAGGTATCTCT
H289P	ACCCAGTGCCCGTGTCGGAG	CCAGACCAGGCCAGGTATCTCTGAC
H289I	AATCAGTGCCCGTGTCGGAG	CCAGACCAGGCCAGGTATCTCT
H289V	AGTCAGTGCCCGTGTCGGAGC	CCAGACCAGGCCAGGTATCTCTGAC
H289T	AACCAGTGCCCGTGTCGGAG	CCAGACCAGGCCAGGTATCTCTGAC
H289Y	ATACAGTGCCCGTGTCGGAGCC	CCAGACCAGGCCAGGTATCTCTGAC
R292A	GCTGTCGGAGCCGCGCGAGAT	GGCACTGTGTCCAGACCAGGCCAG
R292H	CATGTCGGAGCCGCGCGAGAT	GGCACTGTGTCCAGACCAGGCCAG
R292L	CTTGTCGGAGCCGCGCGAGATAT	GGCACTGTGTCCAGACCAGGCCAG
W315L	GCTTGACCAATGTAAATATTGTC	ACCAGCTTGCATGATCTCCG
W315M	GGCATGACCAATGTAAATATTGTC	ACCAGCTTGCATGATCTCCG

### Tissue culture

All cell lines, including INS-1 Ad293, CHO, HeLa, Pt67, SHG44, and PC12 were propagated in Dulbecco's Modified Eagle Medium (Life Technologies, Rockvile, MD) supplemented with 10% fetal calf serum (FCS, HyCLONE, Logan, UT), 2 mM glutamine, 50 U/ml of penicillin G sodium and 50 μg/ml of streptomycin sulfate (Beyotime, Haimen, China). Cells were cultivated in humidified incubators containing 5% CO_2_ at 37°C.

### Transfection of cells

DNA (up to 48 fmol) and 1 μl of polyfect reagent (Qiagen, Shanghai, China) were diluted with serum-free DMEM to 30 and 20 μl, respectively. Diluted polyfect reagent was then added into DNA, votexed, and incubated at room temperature for 10 min. After adding 100 μl culture medium, the DNA mixture (150 μl) was put into a 96-well (Costar, Corning, NY), which had been pre-seeded with 5000 cells overnight. After 12 h transfection, the DNA mixture was removed and fresh culture medium was replenished. Transfections were performed in triplicate and experiments were repeated at least three times. Cells were visualized at 24 h and 48 h using a fluorescence microscope (Eclipse TE 2000, Nikon, Japan) equipped with a CCD camera.

### Flow cytometry analysis

Cells were harvested from 96-well plates after 24 h or 48 h transfection. Samples from three 96 wells were pooled and applied to a BD FACSAria (BD, Franklin lakes, NJ) equipped with a 70 mm nozzle. The instrument was activated with a single argon ion laser (488 nm) and compensated by running an automatic program. Dead cells and debris were excluded. A total of 10,000 events for each sample were acquired and analyzed with FlowJo 7.6.1 (FlowJoChina, Hangzhou, China).

### Measurement of Cre transcripts

The procedure has been described previously with modifications [22)]. Total RNA was isolated from Ad293 and INS-1 cells transfected with pRIP-Cre or pCMV-Cre for 48 h. After reverse transcription, the cDNA was diluted with H_2_O (Dnase and Rnase free, Toyobo) into a volume of 100 μl, of which 5 μl was used for PCR amplification of Cre and 18S rDNA transcripts using primers F4/R4 and F5/R5, respectively.

### Immunofluorescence cell staining

The procedure has been described previously with modifications [22]. Ad293 and INS-1 cells in a 96-well plate were transfected with pRIP-Cre or pCMV-Cre for 48 h. Cells were then fixed with 4% paraformaldehyde for 15 min at room temperature, and permeabilized with 0.2% triton-X 100 for 10 min. Cells were incubated with the Goat anti-Cre antibody (1:100, Santa Cruz, California) for 2 h, and Cy3-Donkey-anti-Goat IgG (1:200, Beyotime, Haimen, China) for 1 h, and finally with DAPI (100 ng/ml, Beyotime, Haimen, China) 5 min. Three PBS washings were performed between each step. Images were taken under a fluorescence microscope equipped with a CCD camera (Nikon, Eclipse TE 2000, Japan).

### Purification of recombinant Cre recombinase

DNA (3 μg) and 10 μl of polyfect reagent (Qiagen, Shanghai, China) were used to transfect cells in 6-well plates (4x10^5^/well, pre-seeded over night) as described above. After 48 h transfection, cells were lysed with 200 μl/well of RIPA buffer (Beyotime, Haimen, China) supplemented with 1 mM pheylmethylsulfonyl fluoride (PMSF, Beyotime, Haimen, China). Protein immunoprecipitation was performed according to the procedure provided by the company (Thermo Scientific, Waltham, MA). The supernatant from four 6-wells was collected by centrifugation at 12,000 g for 5 min, and then applied to a chromatography column pre-filled with Goat anti-Cre antibody (Santa Cruz, Dallas, TX) conjugated resin. After washing three times with Coupling buffer, Cre protein was eluted with 100 μl of Elution buffer. The verification and quantification of Cre protein was performed by Western blotting.

### Western blotting

Purified Cre protein and Cre standard were run in a 10% SDS PAGE gel. After electrophoresis, the proteins were electo-transferred onto a PVDF membrane (MultiSciences, Hangzhou, China), blocked in 2% bovine serum albumin (BSA) solution for 30 min, incubated with Goat anti-Cre antibody (1:1000, Santa Cruz, Dallas, TX) overnight at 4°C. After washing, the membrane was incubated with horse radish peroxidase (HRP)-conjugated Rabbit anti-Goat antibody (Zymed Laboratories, South San Francisco, CA) for 2 h, and finally being developed with ECL (Pierces, Haimen, China) solution for about 30 min. Protein band intensity was quantified by ImageJ software (NIH, Bethesda, MD).

### Measurement of Cre activity *in vitro*

Cre recombination activity *in vitro* was measured according to the procedure provided by the company (NEB, Ipswich, MA). DNA substrate (125 ng) was incubated with various amount of purified Cre protein at 37°C for 30 min, and then analyzed by electrophoresis on 2% agarose gel. DNA band intensity was quantified by ImageJ software (NIH, Bethesda, MD), and used for calculation of the percentage of DNA recombination.

### Statistical analysis

All data were presented as the mean value ± standard deviation (SD) of each group. The student’s t-test was carried out by using GraphPad Prizm 5. *p* values less than 0.05 were considered statistically significant.

## Results

### Substantial non-specific labeling of Ad293 cells by the one-plasmid system

**Fig 2:**
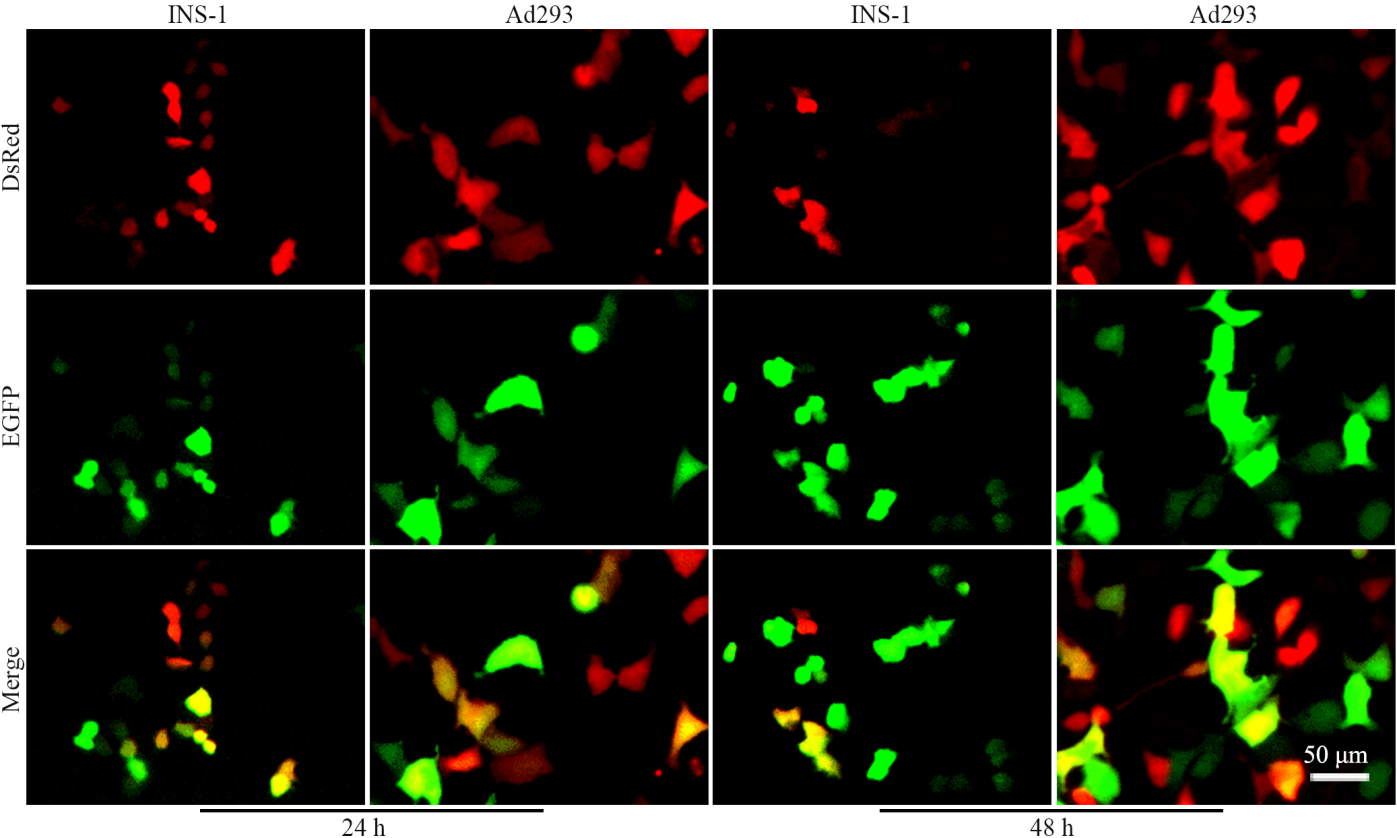
Non-specific labeling of insulin-negative cells by RIP-Cre in a one-plasmid configuration. INS-1 and Ad293 cells (1x10^4^/well) were seeded in a 96-well plate overnight and transfected with 32 fmol/well of DNA (Fig 1A). Cells were observed under a fluorescence microscope. Images were taken with a CCD camera. Micrographs show the expression of DsRed (upper panel), EGFP (middle panel), and Merge (lower panel) in INS-1 and Ad293 cells after 24 and 48 h transfection.

We constructed the RIP-Cre and reporter expression cassettes in a one-plasmid and two-plasmid systems (Fig 1). Cre recombinase is supposedly expressed only in insulin producing cells due to the regulation of tissue-specific rat Ins2 promoter, which then in turn results in deletion of the floxed DsRed sequence and activation of EGFP. Thus if the system displays strong specificity, insulin producing cells should be green while non-insulin producing cells should be red. However, when the one-plasmid system (Fig 1A) was used in the transfection, EGFP signals not only appeared in insulin-positive INS-1 cells, but also found frequently in insulin-negative Ad293 cells (Fig 2).

**Fig 3:**
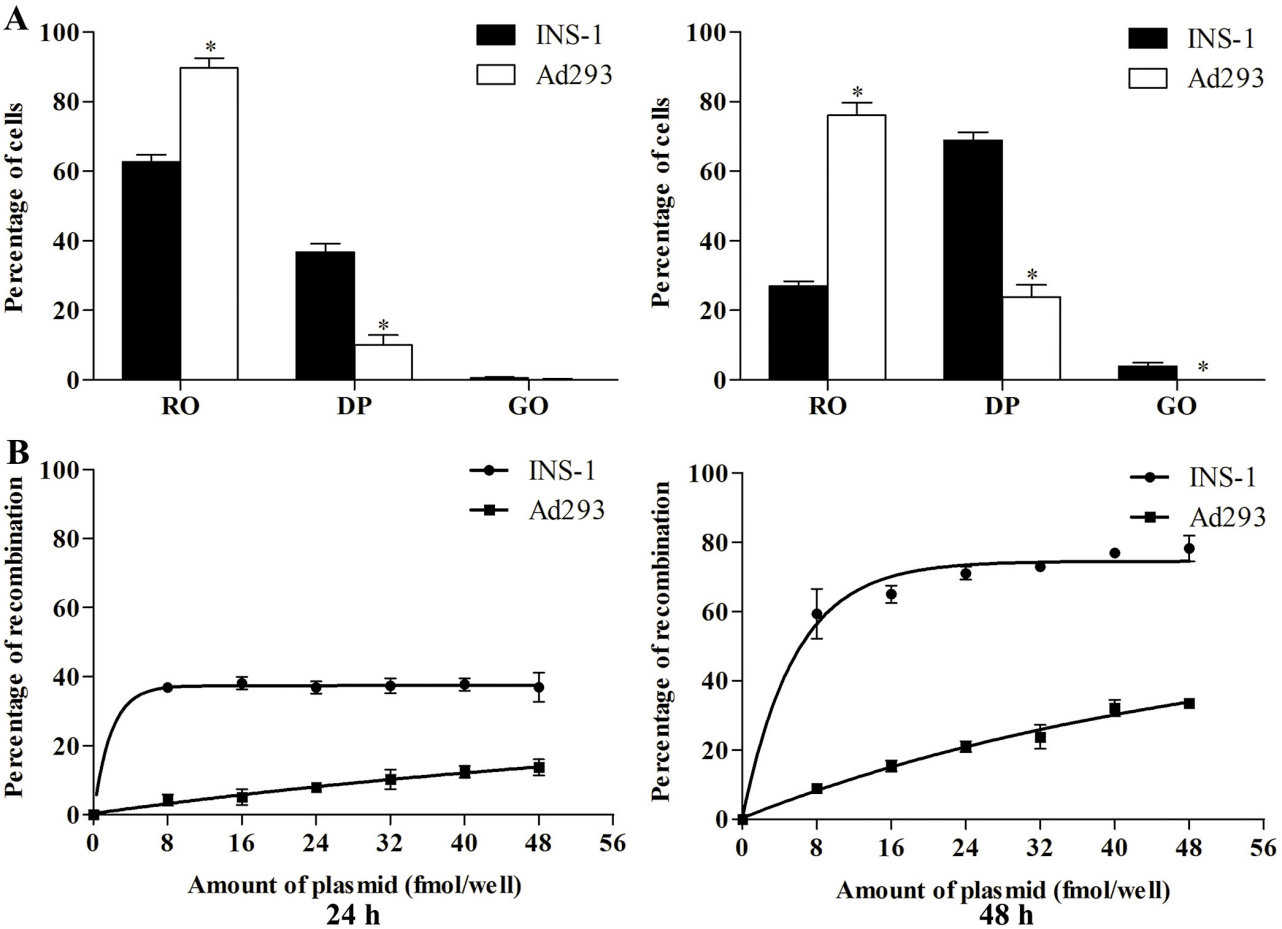
Quantitative analysis of the percentage of recombination by RIP-Cre in a one-plasmid configuration. The experiments were performed as described above with 32 fmol/well of DNA (A), or a concentration gradient of DNA (B). Cells from triplicate were pooled at 24 h (left) and 48 h (right), and analyzed by FACS. The percentage of DsRed+ only (RO), DsRed+/EGFP+ (DP), and EGFP+ only (GO) Cells (A), and the percentage of recombination (% of EGFP+) (B) were calculated using the total number of fluorescence positive cells as the denominator. N = 9. (*p<0.05, as compared to its immediate counterpart to the left).

Quantitative analysis by FACS showed that the percentage of EGFP+ Ad293 cells increased from about 10% after 24 h transfection to approximately 20% at 48 h (Fig 3A). On the other hand, EGFP+ INS-1 cells reached almost 40% and 80%, respectively, after 24 and 48 h transfection. Most of the EGFP+ cells were also DsRed+, few EGFP-only cells appeared at 48 h. Increasing the amount of DNA apparently saturated the recombination efficiency quickly in INS-1 cells, but the percentage of EGFP+ Ad293 cells increased accordingly (Fig 3B). It is noteworthy that, even with minimum amount of DNA, below which transfection of INS-1 cells was not possible, the non-specific targeting in Ad293 cells was still substantial.

### The rat Ins2 promoter is tightly controlled *in vitro*

**Fig 4:**
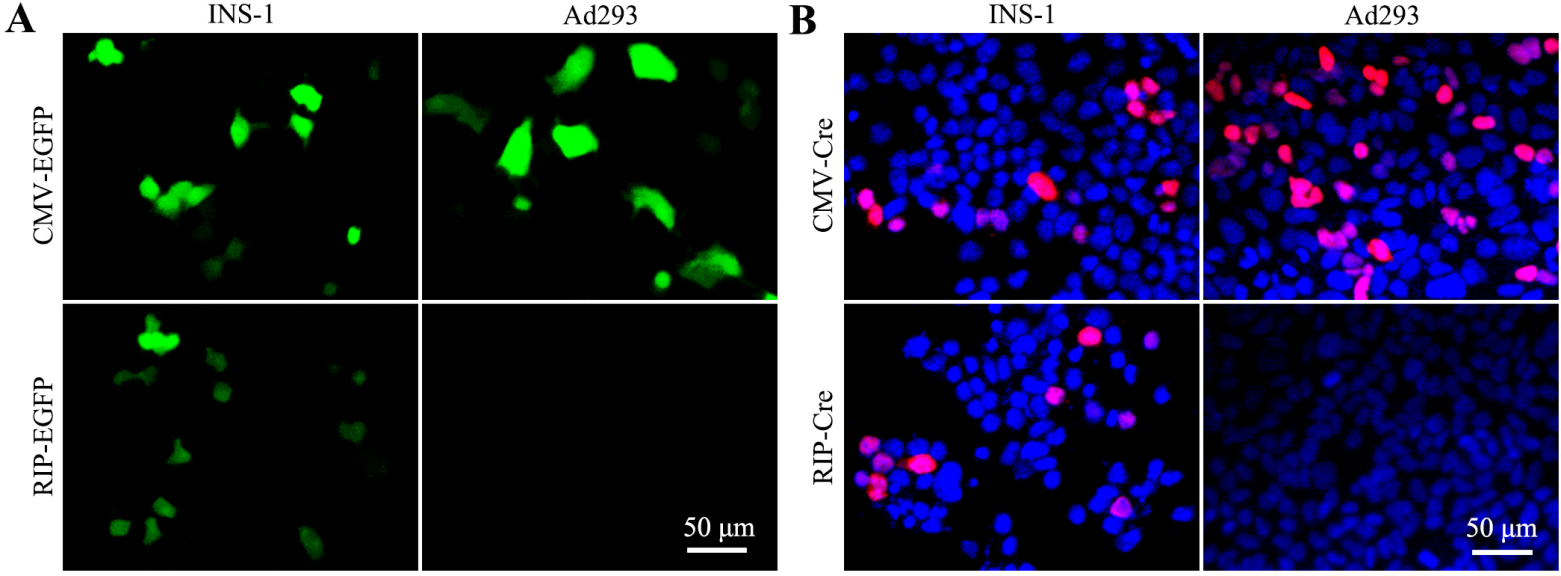
Verification of the tissue specificity of RIP. INS-1 and Ad293 cells (1x10^4^/well) were seeded in a 96-well plate overnight and transfected with 32 fmol/well of indicated plasmid (Fig 1C). (A) Micrographs show the expression of EGFP in INS-1 and Ad293 cells after 48 h transfection. (B) Cre protein was revealed by immunofluorescence staining.

One reason to explain the observation of EGFP signal in insulin-negative cells is the lack of tissue specificity of RIP used in this study. To test this hypothesis, we put the EGFP gene under the control of RIP (Fig 1C) and performed transfection experiment. EGFP signal was completely absent in Ad293 cells transfected with pRIP-EGFP, but robustly measurable in INS-1 cells (Fig 4A)

Further, we performed immunofluorescence staining to detect Cre expression in Ad293 and INS-1 cells transfected with pRIP-Cre (Fig 1B). The result showed that Cre was primarily located in the nucleus of INS-1 cells, but obviously absent in Ad293 cells (Fig 4B). Consistently, Cre transcripts identified by RT-PCR were found only in INS-1 cells, but not in Ad293 cells, after 48 h transfection (Data not shown).

### Non-specific targeting was also found with the two-plasmid system

**Fig 5:**
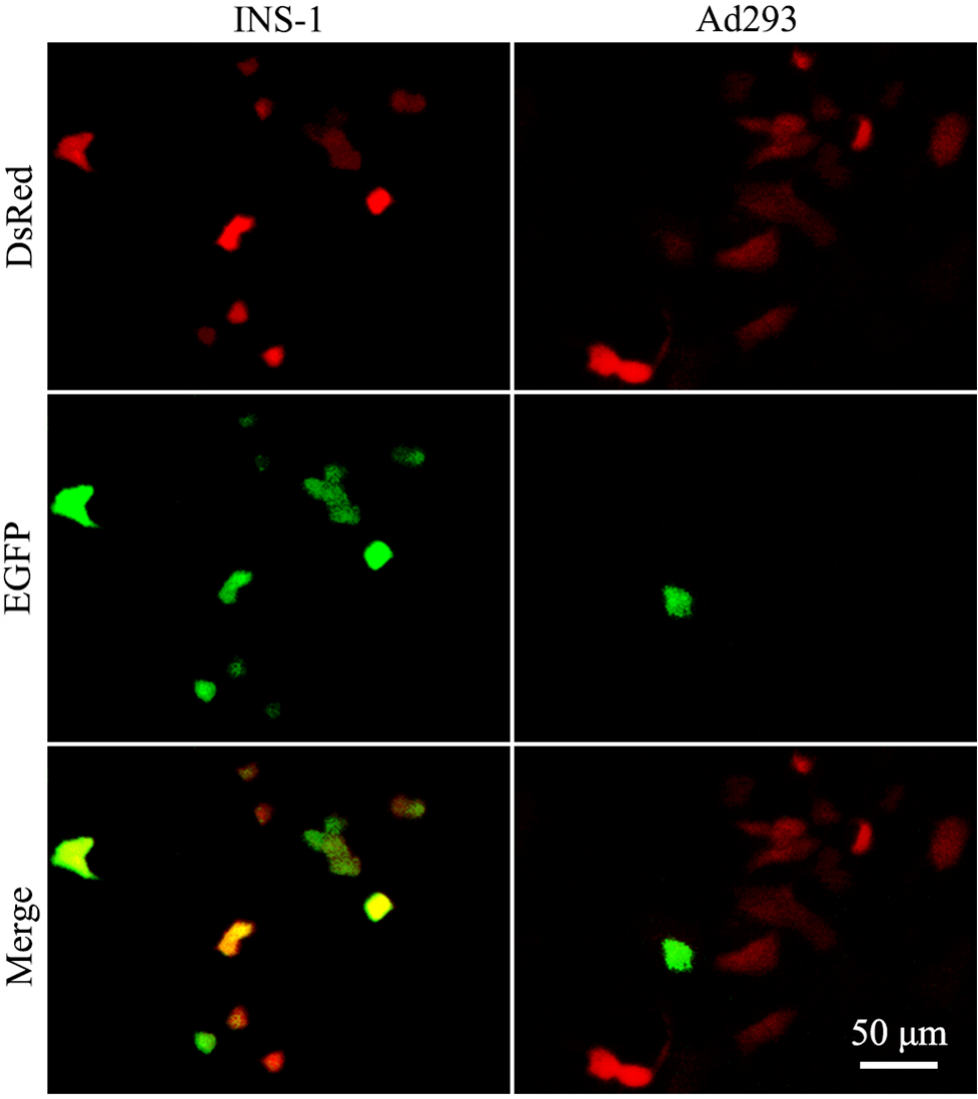
Detection of promoter interference between CMV and RIP. INS-1 and Ad293 cells (1x10^4^/well) were seeded in a 96-well plate overnight and transfected with 32 fmol/well of plasmid DNA for promoter interference (Fig 1D). Micrographs show the expression of DsRed (upper panel), EGFP (middle panel), and Merge (lower panel) in INS-1 and Ad293 cells after 48 h transfection.

Another possible explanation is that the CMV promoter deteriorates the tissue specificity of RIP. We therefore put EGFP and DsRed under the control of RIP and CMV, respectively, and constructed into a single vector (Fig 1D). When the plasmid was transfected into Ad293 cells, very little EGFP signal was detected (Fig 5). Increasing the amount of DNA did not affect the extent of EGFP expression in Ad293 cells (Data not shown), indicating that promoter interference was almost negligible.

**Fig 6:**
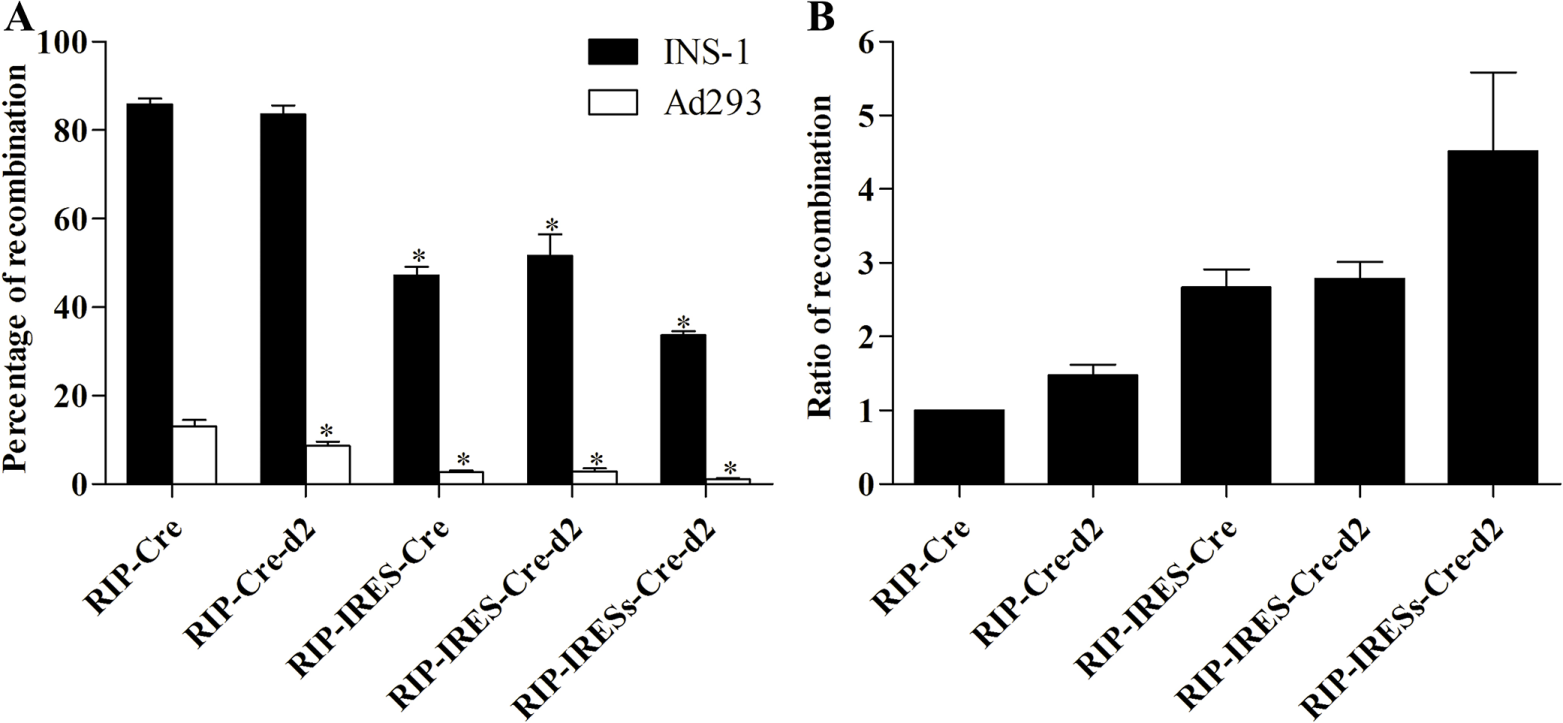
Increased specific labeling of insulin-positive cells by reducing the translation level of Cre in a two-plasmid configuration. INS-1 and Ad293 cells (1x10^4^/well) were seeded in a 96-well plate overnight and transfected with indicated plasmids (32 fmol of each plasmid/well) (Fig 1E). Cells from triplicate were pooled at 48 h and analyzed by FACS. The percentage of recombination (A), and the ratio of recombination (B) between INS-1 and Ad293 cells were calculated. N = 9. (*p<0.05, as compared to that of RIP-Cre).

To formally evaluate the effect of CMV on RIP, we generated a two-plasmid system, one for the RIP-Cre cassette, and the other for the reporter construct CMV-loxP-DsRed-loxP-EGFP (Fig 1B). Still, recombination in Ad293 cells accounted about 15% after 48 h transfection (Fig 6A), demonstrating that promoter interference was not the major factor underpinning the RIP non-specificity.

### Reduced expression of Cre protein levels increased labeling specificity

The above result also suggested that although Cre expression was extremely low in Ad293 cells transfected with pRIP-Cre, whereas the trace amount of enzyme still had high enough activities to produce recombination between loxP sites. If that was the case, reducing the expression levels of Cre would probably suppress the recombination in insulin-negative cells.

To achieve this goal, we fused the murine PEST fragment of the ornithine decarboxylase (MODC, d2) to the C-terminus of Cre and thus creating a quick degrading Cre-d2 (Fig 1E). However, no effect on the recombination in Ad293 cells was observed (Fig 6A). We then introduced the internal ribosomal entry site in front of the Cre recombinase gene in frame (IRES) or out-of-frame (IRESs), and thus reduced the translation efficiency (Fig 1E). This arrangement resulted in significantly reduced recombination in Ad293 cells (Fig 6A), although very few EGFP+ cells were still identifiable. As expected, the recombination in INS-1 cells also reduced to a certain degree; however, the ratio of recombination in INS-1 *v*.*s*. Ad293 cells increased 3-4-fold (Fig 6B), revealing an increase of RIP-Cre specificity.

### Enhanced specific targeting could be achieved by using Cre mutants

An alternative approach is to reduce Cre activity without actual decrease in protein amount. Eighteen Cre variants with point-mutation were constructed. The targeted amino acids are conserved in members of the tyrosine recombinase family of site-specific recombinases and important for the catalytic activity [23].

**Fig 7:**
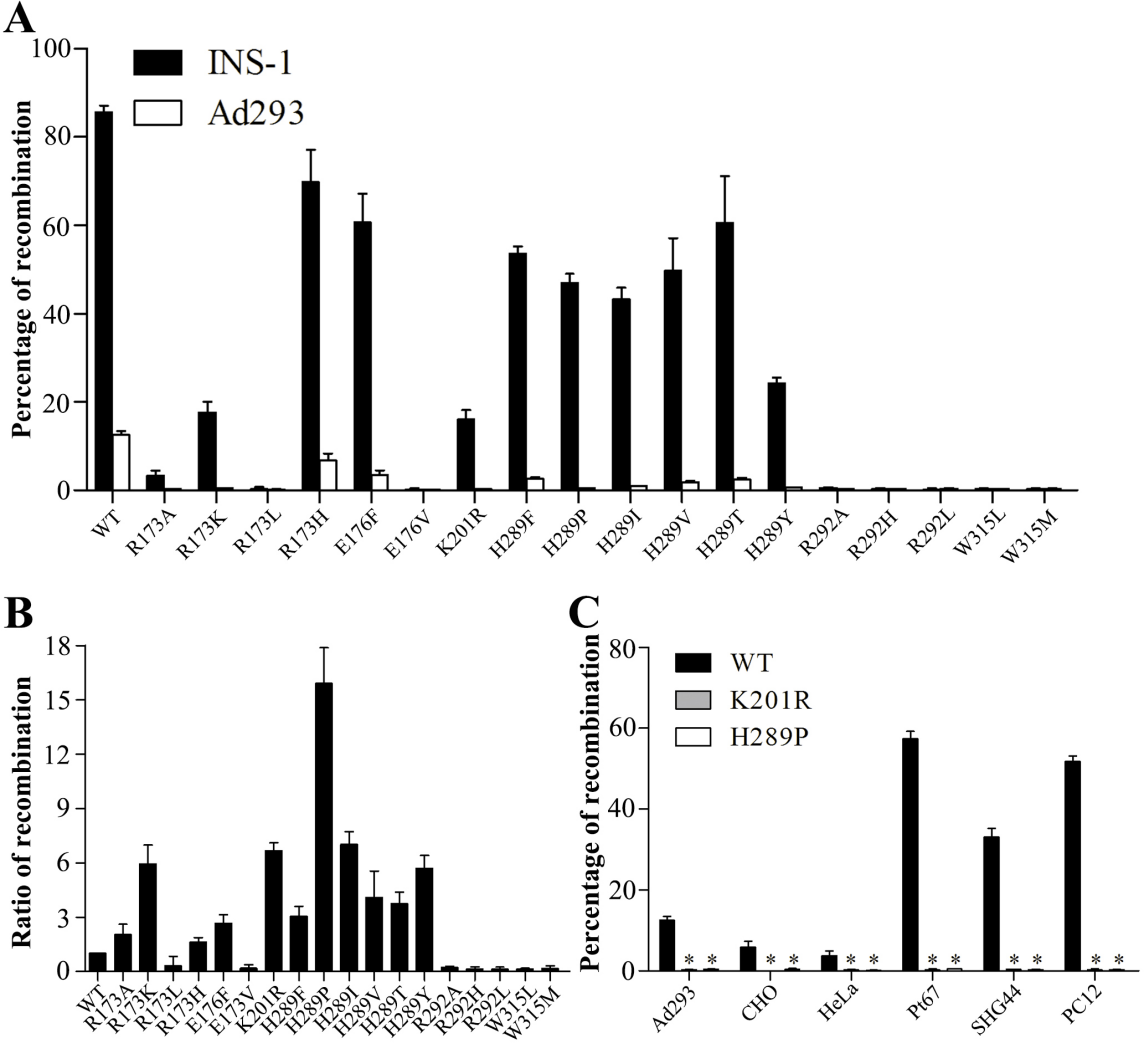
Increased specific labeling of insulin-positive cells by reducing the enzymatic activity of Cre in a two-plasmid configuration. INS-1 and Ad293 cells (A, B) and CHO, HeLa, Pt67, SHG44 cells (C) (1x10^4^/well) were seeded in a 96-well plate overnight and transfected with indicated plasmids (32 fmol of each plasmid/well) (Fig 1B). Cells from triplicate were pooled at 48 h and analyzed by FACS. The percentage of recombination in insulin-negative cells (A, C) and the ratio of recombination between INS-1 and Ad293 cells (B) were calculated. N = 9. (*p<0.05, as compared to that of wild type Cre).

All mutants had reduced recombination in both INS-1 and Ad293 cells (Fig 7A). However, the ratio of recombination between INS-1 *v*.*s*. Ad293 cells increased up to about 15-fold as compared to that of the wild type (Fig 6B). We chose Cre(K201R) and Cre(H289P), which showed negligible recombination activity in Ad293 cells but substantial in INS-1 cells, for further study. Wild-type Cre generated various but significant proportion of EGFP signals in insulin-negative Ad293, CHO, HeLa, Pt67, SHG44 and PC12 cells, whereas neither Cre(K201R) nor Cre(H289P) produced detectable EGFP+ cells (Fig 7C). Moreover, when both mutants were configurated into a one-plasmid system, no EGFP+ cells were identified in insulin-negative cells (Data not shown).

**Fig 8:**
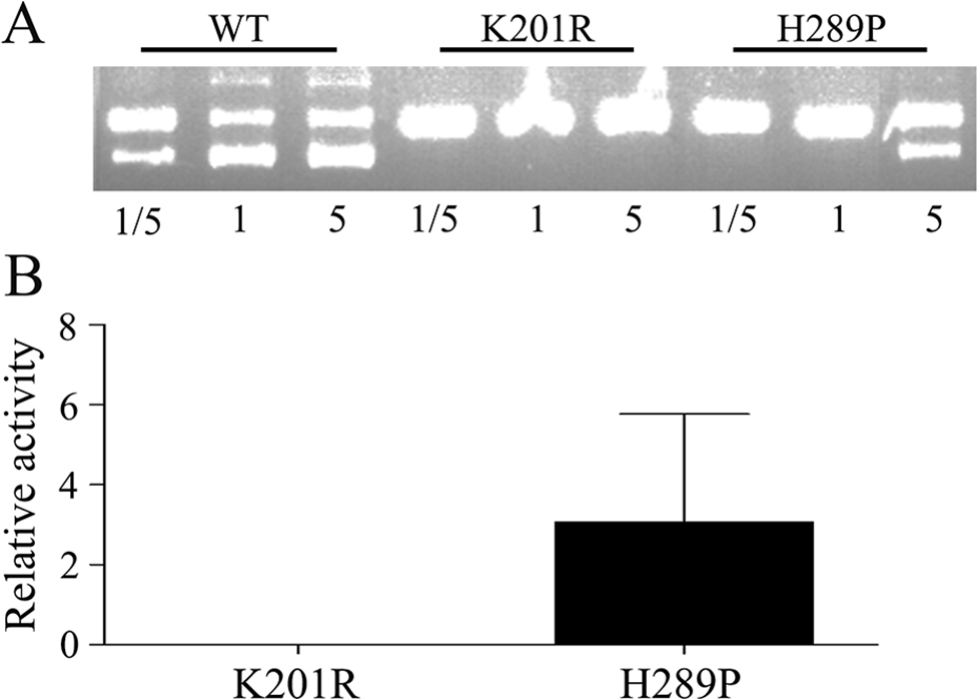
Measurement of Cre activity in an *in vitro* DNA digestion assay. (A) DNA substrate (125 ng) was incubated with various amount (1/5, 1, or 5 μl) of IP-purified wild-type Cre (WT), or CreK201R, and CreH289P protein at 37°C for 30 min. (B) Relative activity of CreK201R and CreH289P as compared to the wild type was estimated based on the intensity of the DNA bands, which was then normalized to the amount of Cre protein determined by Western Blot analysis (Data not shown). The experiment had repeated twice.

Finally, *in vitro* DNA substrate digestion assay was carried out to measure the enzymatic activity of Cre protein. In consistent with the *in vivo* recombination capability, the activity of Cre(H289P) *in vitro* reduced to about 3% as compared to that of wild-type (Fig 8). No enzymatic activity *in vitro* was detected for Cre(K201R).

## Discussion

Since the first introduction of the RIP-Cre mouse line [24], where RIP represents a short (∼0.6 kb) fragment of the rat Ins2 promoter, more than a dozen similar mice have been created and used to delete genes or activate reporters in pancreatic beta-cells [1,12], revealing new processes and functions that can be imperative for finding a cure to diabetes.

The major caveat of these RIP-Cre transgenic lines lies in the noticeable ectopic expression of Cre in the brain [10–14]. Since RIP encompasses only a short segment of the intact rat Ins2 promoter, it is conceivable that the specificity might be mitigated. However, genes knocked into the endogenous rat Ins2 locus still showed clear expression in multiple regions of the brain [25,26]. Therefore, the data possibly reflects a resemblance in transcriptional control during development between the endocrine pancreas and the neuronal system [27–29].

In the present study, we employed a sensitive in vitro assay for measurement of RIP-Cre labeling specificity. Indeed, robust non-specific recombination was detected in all tested insulin-negative cells, including Ad293, CHO, HeLa, Pt67, SHG44 and PC12 (Figs 2 and 7C). The recombination was truly Cre-loxP-mediated as demonstrated by the PCR analysis on plasmid recovered after transfection (Data not shown). The non-specific recombination could not be explained by the lack of specificity of RIP, as neither Cre transcripts nor proteins were detected in Ad293 cells (Fig 4). In addition, the phenomenon of non-specific labeling could not be accounted by promoter interference [30), as only occasional EGFP signal was detected in Ad293 cells transfected with pCMV-DsRed-RIP-EGFP (Fig 5). Since a polyadenylation site was placed between DsRed and RIP, this configuration could have reduced the probability of promoter interference [31].

Further, rather than focusing on the improvement of insulin promoter specificity [32], we took on two alternative approaches to increase RIP-Cre specific labeling. One approach exploited the IRES to reduce the translation efficiency, and thus decreases the protein levels, resulting in more than 4-fold increase in ratio of recombination between INS-1 and Ad293 cells (Fig 6). On the other hand, we generated more than a dozen point mutations targeting conserved amino acids of the catalytic domain of Cre [23,33]. One of the mutants, Cre(H289P), had the most profound effect on RIP specificity, showing more than 15-fold increase in ratio of recombination between INS-1 and Ad293 cells (Fig 7A and 7B). Also, Cre(H289P) displayed a reduction of enzymatic activity by about 30-fold as compared to that of wild-type (Fig 8), which is consistent to a previous report [33]. It should be point out that the EGFP signals in Ad293 cells transfected with pRIP-Cre(H289P) were not significantly above background levels. In fact, when inspected manually under fluorescence microscope, none EGFP+ Ad293 cell was identifiable from a total of more than 1000 DsRed+ cells. At the same time, the recombination efficiency in INS-1 cells maintained at about 50%, which constitutes a 35% reduction from that of wild type Cre. The results demonstrated that Cre(H289P) was superior to IRES in terms of increasing specificity while maintaining labeling efficiency of RIP-Cre (Figs 6B and 7B).

It is noteworthy that the non-specific recombination in fibroblastic Pt67, SHG44 and PC12 cells was much higher than that in epithelial cell types including Ad293, CHO, and HeLa cells (Fig 6C), implying that RIP activity varied dramatically even under suppressive environments. Taking into the consideration of the neuronal origin of SHG44 and PC12 cells, the result is in consistent with the finding that ectopic expression of Cre was predominantly found in the brain [10–14]. Nonetheless, when pRIP-Cre(H289P) was used, non-specific labeling reduced to background levels, demonstrating that deceasing the activity of Cre to a certain degree can be an effective solution to increasing RIP-Cre specificity.

On the other hand, when a long mouse Ins1 promoter was used to produce the MIP1-GFP mice, GFP expression was found confined to the pancreatic islets only [34], reflecting that mouse Ins1 promoter is relatively weaker than rat Ins2 promoter [35]. However, when treated with streptozotocin, resulting in beta-cell apoptosis and thus diabetes, GFP signals were found in the liver, adipose tissue and bone marrow [16]. Therefore, finding a highly specific promoter for islet beta-cells seems intrinsically difficult, because absolutely tissue-specific cis-acting regulatory elements probably do not exist in nature. In this regard, neither the Pdx1 nor the Ngn3 promoter was active solely in the endocrine pancreas [1,14,36]. Nonetheless, we also cloned a 1.1 kb fragment of mouse Ins2 promoter (MIP2), and found that pMIP2-Cre behaved similarly to pRIP-Cre (Data not shown), confirming that the lack of absolute tissue specificity was not restricted to RIP.

The inducible version of Cre (CreER), created by fusion with a modified estrogen receptor that, in the presence of tamoxifen, is able to translocate from the cytoplasm to nucleus to cleave the loxP tagged DNA. Similarly, ectopic expression of Cre activity has been reported in RIP-CreER and Pdx1-CreER mouse strains [14]. In addition, unintended recombination can occur in the absence of tamoxifen in older mice [37]. Although the “leakiness” can be reduced to a reasonable level by introducing mutations to the ER domain [1,38], it still raises serious concerns about the interpretations of the data. Recently, a MIP1-CreER line was created using an 8.3 kb fragment of the mouse Ins1 gene promoter [39]. CreER mRNA expression was not detected by qRT-PCR in a panel of non-pancreatic tissues. However, its absolute specificity to the islet beta-cells needs further examination, given that extrapancreatic GFP expression was detected in diabetic MIP1-GFP mice [16]. In this regard, our preliminary data showed that un-induced recombination was completely eliminated by incorporating H289P mutation into CreER (Data no shown), demonstrating a wilder application of this approach.

One last, but not the least, potential beneficial effect of Cre(H289P) could be the reduced toxicity, which has been reported in some mammalian cells expressing high levels of the wild type Cre recombinase [17,40,41], possibly due to recombination between cryptic loxP sites in the mammalian genome [19]. Genome perturbation and damage have also been postulated to compromise beta-cell function in RIP-Cre mice, leading to glucose intolerance and partial impairment in insulin secretion [17,18]. It is conceivable that off-target recombination between cryptic loxP sites will be significantly reduced by using Cre(H289P) with decreased activity. Further, such a strategy might be also suitable to reducing off-target recombination in other genome editing systems, such as ZFNs [42,43], TALENs [44–46] and Crisper/Cas9 [47,48].

In summary, in the present study, we offered two approaches, one reduces the protein level and the other decreases the enzymatic activity, for improved specific labeling in INS-1 pancreatic beta-cells with RIP-Cre. However, the applicability of the approach needs to be tested further in *in vivo* study. Although a reasonable tradeoff in labeling efficiency is inevitable, the approach could be important to the study of islet biology and the treatment of diabetes in the future.
